# Determining and Prioritizing the Organizational Determinants of Emergency Medical Services (EMS) in Iran

**DOI:** 10.5812/ircmj.2192

**Published:** 2013-04-05

**Authors:** Mohammadkarim Bahadori, Ramin Ravangard

**Affiliations:** 1 Health Management Research Centre, Baqiyatallah University of Medical Sciences, Tehran, IR Iran; 2 School of Management and Medical Information Sciences, Shiraz University of Medical Sciences (SUMS), Shiraz, IR Iran

**Keywords:** Organizations, Emergency Medical Services, Iran

## Abstract

**Background:**

Improving the organization of pre-hospital emergency to provide emergency medical services (EMS), as a part of health system, plays an important role in timely and properly response to incidents, as well as, reducing mortalities and disabilities.

**Objective:**

This study was conducted to determine the organizational determinants of emergency medical services in Iran and analyze their relationship and prioritize them.

**Materials and Methods:**

The present study is kind of descriptive and cross-sectional study that has been conducted on the first half of 2010 using DEMATEL method (a group decision-making technique). Required data were collected using a questionnaire from a sample of 30 Iranian experts in pre-hospital emergency, who were selected using available sampling method.

**Results:**

The determinants of establishing an independent EMS organization as a policy maker and observer organization, providing services through public organizations such as Emergency 115, private organizations partnership in pre-hospital emergency system, and integrating pre-hospital and hospital emergency under single supervision and management were determined as organizational determinants. Also, establishing an independent EMS organization and integrating pre-hospital and hospital emergency under single supervision and management were determined as the most affecting and affected organizational determinants, respectively, with the coordinates (1.01 and 1.01) and (0.85 and - 0.85) in the pre-hospital emergency organizational determinants graph.

**Conclusions:**

Emergency medical services should be considered as a system with its independent components. Establishing an independent EMS organization, integrating pre-hospital and hospital emergency under single supervision and management, as well as, extending the possibility of providing EMS through private sector are essential in order to make fundamental reforms in providing emergency medical services in Iran.

## 1. Background

Addressing the human health issues is a high priority in every civil society. Given the increasing number of emergency patients, it is very important to address such problems because of living in a riskyenvironment and hazardous circumstances. (1). One of the main concerns of the authorities of our society is to address the health and curative issues, especially the treatment of emergency patients, in a proper and timely manner. In other words, everyone concerns about the receiving pre- and in-hospital required care by needy patients (2). Therefore, the improvement of pre-hospital emergency organization to provide emergency medical services (EMS), as a part of health system, plays an important role in timely and properly response to incidents, as well as, reducing mortalities and disabilities (3). It is also important, because of the change of diseases pattern from contagious diseases to non-communicable ones, and the increase in traffic accidents and cardio-vascular diseases in Iran. Although some considerable measures have been taken to improve pre-hospital indicators in Iran during last years, there is still a long distance from international standards and there isn't any significant decrease in mortalities compared with those of previous years. However, it is noticeable in the pre-hospital emergency medical services that more that 75% of mortalities occur in the scene and during transporting injured to a hospital (4). Those countries which have had a future-driven view about EMS, have been successful in improving pre-hospital emergency organization so that they allocate their available resources suitably and properly through proper planning in crisis situations. In these countries, emergency medical services are considered as a complete system with its independent components. These components interact with each other and result in an efficient system (5-7). EMS in many developed countries reach scene and provide medical services and transport them using advanced air and land systems, equipped communication and telecommunication networks, experienced staff, providing needed training, and required inter- and intra-section coordination (8, 9). The majority of studies conducted in the previous years in Iran have focused more on the emergency services performance, the quality of patient transportation, patient satisfaction from emergency medical services, and other issues related to emergency units. However, since 2010, valuable researches have been conducted by researchers in conjunction with pre-hospital emergency development (10, 11). In developed countries, many researchers have described the current status of their country EMS, and their experience can be used by developing and underdeveloped as well as less developed countries. The common feature of all of these studies is the consideration of the hospital and pre-hospital emergency medical services as emergency medical services subcategories, so that they have well explained the emergency medical services components such as their organization, human resources, transportation, education and training, communication and processes (6, 8, 12). Haghparast et al. in their study on facilities and obstacles to provide trauma care for emergency patients in Iran in 2010, concluded that the most important EMS components were their organization and management, qualified manpower and staff, availability of needed recourses, and communication (10). According to Zargar's study on trauma care system conducted in 2011, the most important components of trauma care in Iran were their managerial, clinical and operational components, as well as, final care facilities and evaluation (11). The results of a study on the differences between developed and developing countries emergency medical services systems conducted by Roodsari et al. in 2006 showed that there should be some international attempts to set a series of minimum standers on emergency patients care (13). Considering the high rate of traffic accidents in Iran, related authorities should take equipping and evolving emergency medical services network into consideration as a high priority issue in the health system planning.

## 2. Objective

This study was conducted to determine the organizational components of the emergency medical services in Iran and analyze their relationship and prioritize them.

## 3. Materials and Methods

The present study is kind of descriptive and cross-sectional study that was conducted on the first half of 2010 using DEMATEL method (a group decision-making technique). The organizational determinants of emergency medical services in different systems were collected and a questionnaire was developed. Afterwards, a sample of 30 Iranian experts in pre-hospital emergency, who were selected using available sampling method, were asked to evaluate the suggestions and revise the collected determinants using their scientific, practical and visionary experience and all of them were faculty member. The choice criteria of these selected experts were being academic and having experience or administrative responsibilities in emergency medical services. The determinants were identified using Delphi method. Then, the Influential determinants were determined using SPSS 17.0 software (One-Sample T-Test) and the experts were asked to indicate the relationship among those determinants and, finally, the related graph was depicted using MATLAB and Visio software. The DEMATEL technique was developed by Battelle Memorial Institute Research Program during 1972-1976 to study and solve the complex issues. The reason behind the development of DEMATEL technique was that the proper use of scientific research methods could improve the complicated structure of issues and help recognize and choose the practical solutions with hierarchical structures. DEMATEL technique is based on oriented graphs diagraphs which can divide effective components into two groups, cause and effect. These diagraphs depict the dependency relationship between the components of a system. Causal diagraphs are obtained through regular pairs (DK + RK,DK - RK) in which the horizontal (D + R) and vertical (D - R) axes called, respectively, the "prominence" -can be made by adding DK to RK-, and the "relation" -can be made by subtracting RK from DK. If the quantity of (DK-RK) is positive, that criterion will relate to the cause group and if it is negative, the criterion will relate to the effect group. Therefore, the causal diagraphs can convert complex causal relationships among components into a visible structural model and provide an accurate insight for resolving the considered issues. Furthermore, the right decisions can be made using causal diagrams and recognizing the differences between cause and effect criteria (14, 15).

## 4. Results

According to the initial findings of this research, establishing an independent EMS organization as a policy maker and observer organization, independent deputy for EMS in Medical Sciences Universities, providing services through public organizations such as Emergency 115, private organizations partnership in pre-hospital emergency system, providing services through voluntary organizations (such as non-governmental organizations, charities, etc.) and integrating pre-hospital and hospital emergency under single supervision and management were suggested. However, Iranian pre-hospital emergency experts agreed with all of these pre-hospital emergency organizational determinants except independent deputy for EMS in medical sciences universities and providing services through voluntary organizations (such as non-governmental organizations, charities, etc.) (P = 0.001) (Table 1).

**Table 1.** The Results of the Expert Opinions about Pre-Hospital Emergency Organizational Components in Iran

Components	Expert Responses							
	Completely Agreed	Agreed	Without any Responses	Opponent	Completely Opponent	Mean	SD	P Value
**Establishing an independent EMS organization as a policy maker and observer**	22	8	0	0	0	4.73	0.44	0.0001
**Independent deputy for EMS in Medical Sciences Universities**	3	1	5	9	12	2.13	1.27	0.0001
**Providing services through public organizations such as Emergency 115**	15	15	0	0	0	4.50	0.5	0.0001
**Private organizations partnership in pre-hospital emergency system**	16	13	1	0	0	4.50	0.57	0.0001
**Providing services through voluntary organizations (such as non-governmental organizations, charities, etc.)**	1	1	5	14	9	2.03	0.96	0.0001
**Integrating pre-hospital and hospital emergency under single supervision and management**	12	18	0	0	0	4.60	0.49	0.0001

**Figure 1.**
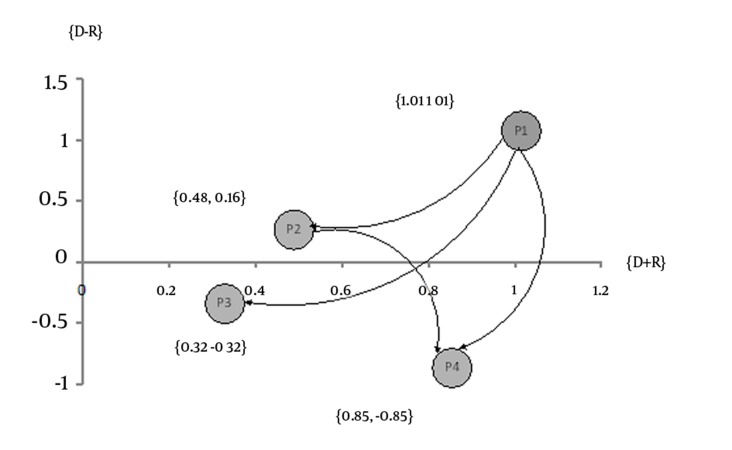
The Prioritizationof Pre-Hospital Emergency Organizational Components in Iran P1: Establishing an independent EMS organization, P2: Private organizations partnership in pre-hospital emergency system, P3: Providing services through public organizations, P4: Integrating pre-hospital and hospital emergency under single supervision and management

Also, the results of this study showed that the components of establishing an independent EMS organization (P1) and private organizations partnership in pre-hospital emergency system (P2) were certainly penetrating the system, which were placed in the cause group as first to second priorities. while the determinants of providing services through public organizations (P3) and integrating pre-hospital and hospital emergency under single supervision and management (P4) were partially influenced , and were placed in the effect group as third to fourth priorities (Table 2 and Figure 1). The determinants of establishing an independent EMS organization and integrating pre-hospital and hospital emergency under single supervision and management were determined as the most affecting and affected organizational determinants, respectively, with the coordinates (1.01 and 1.01) and (0.85 and -0.85) on the pre-hospital emergency organizational determinants graph.

**Table 2.** The Hierarchy of Affecting and Affected Pre- Hospital Emergency Organizational Determinants in Iran

	Determinants			
Components	D	R	D+R	D-R
**Establishing an independent EMS organization (P1)**	1.01	0	1.01	1.01
**Private organizations partnership in pre-hospital emergency system (P2)**	0.32	0.16	0.48	0.16
**Providing services through public organizations (P3)**	0	0.32	0.32	-0.32
**Integrating pre-hospital and hospital emergency under single supervision and management (P4)**	0	0.85	0.85	-0.85

## 5. Discussion

The results of several studies show that the main components of EMS are the care in the accident sites, the care in the patient transportation and the care in the hospital which are related to each other and lack of each one results in inefficient emergency medical services (16) . Emergency care needs accuracy in planning and implementation, and different components of EMS should be related to each other so that the whole system acts as a single unit (17). Based on the findings of current study, the components of the establishing an independent EMS organization as a policy maker and regulator, providing services through public organizations such as Emergency 115, private organizations partnership in pre-hospital emergency system and integrating pre-hospital and hospital emergency under single supervision and management were indicated as influential components on pre-hospital emergency organization. The component of establishing an independent EMS organization is influential on the other components however isn't impressible from others which show its effectiveness on EMS reform and development in Iran. One of the reasons for the success of countries in pre-hospital emergency services is the establishment of an independent EMS organization so that all service providers including the private, public, voluntary, etc. sectors work under its supervision. This prevents from task overlaps and results in all of these sectors follow single policies (6, 18, 19). The results of Van Rooyen, Thomas and Clem's study (1999) showed that the evaluation of emergency medical services systems could be useful for providing models of developing systems including voluntary, private, hospital-based and complex systems which all of these models included organization component as one of influential components on pre-hospital emergency performance. Also, they found that pre-hospital emergency organization as an independent organization which related all of sub-systems to each other played an important role in EMS efficiency and effectiveness. Therefore, organizational features should be completely clear and obvious in providing pre-hospital care models (20). These results, therefore, confirm our study findings. Inappropriate organization and management had been introduced as the main obstacle of providing suitable pre-hospital emergency medical services and improper structure of EMS had been described as one of the most important determinants of organization and the most influential determinant in EMS reform and development in Iran based on Haghparast et al.'s study results. These results are consistent with our study findings (10). The results of another study conducted in Iran showed the lack of coordination among different EMS providers at the point of service and concluded that the establishing an independent EMS organization as a policy maker and observer could help, to a large extent, reduce this inconsistency (21). The Weninger, Hertz and Mauritz's study results, conducted in Austria, showed that the providing EMS was different among its provinces, however, was similar in most of European countries (22). The results of Vaitkaitis' study (2007) on the current status and challenges of Lithuania EMS system showed that one of its most important problems was the absence of integrated pre -hospital and hospital care systems (23). The current study results, also, showed that integrating pre-hospital and hospital emergency under single supervision and management was a priority determinant in EMS reform and development in Iran. Therefore, the findings of these two studies confirm each other. Hunyadi-Anticevic, in a study on the EMS System in Croatia, found that in order to assure the quality of provided care and services, both hospital and pre-hospital emergency medical services should be expanded and integrated. Also, the results showed that the improvement of care occurs when hospital and pre-hospital emergency services were coordinated (24). These results, too, are consistent with our findings. Based on the results of another study, private sector in most developing countries has an important role in providing EMS. In the current study, also, private organizations partnership in pre-hospital emergency system was indicated as one of causal and influential components. Reliance on private sector to provide services - under government supervision and management – is one of the accepted principles of economics and management sciences. However, in some countries including Iran, private sector doesn't have any important and considerable role in providing needed services. Therefore, this sector should be allowed to have major contribution to provide different services including EMS in order to have an efficient pre-hospital emergency medical services system and pursue the policies of 44 principles (25). In conclusion, emergency medical services should be considered as a system with its independent components. Establishing an independent EMS organization, integrating pre-hospital and hospital emergency under single supervision and management, as well as, extending the possibility of providing EMS through private sector are essential in order to make fundamental reforms in providing emergency medical services in Iran.
